# Development and Preliminary Validation of a Multidimensional Questionnaire on Nutrition Knowledge, Food Composition Knowledge, and Dietary Habits in Medical Students

**DOI:** 10.3390/nu18162720

**Published:** 2026-08-20

**Authors:** Ana Presa-Carrera, Eva Luyufeng Yáñez-Izquierdo, Carlos Enrique Adán-Failde, Juan Manuel Vázquez-Lago

**Affiliations:** 1 Department of Preventive Medicine and Public Health, University of Santiago de Compostela, 15786 Santiago de Compostela, Spain; 2 Department of Preventive Medicine, Santiago de Compostela University Teaching Hospital, 15703 Santiago de Compostela, Spain; 3 Health Research Institute of Santiago de Compostela (IDIS), 15706 Santiago de Compostela, Spain

**Keywords:** nutrition knowledge, medical students, dietary habits, food classification, questionnaire validation, psychometrics, nutrition education, public health nutrition

## Abstract

**Background/Objectives**: Nutrition is an important component of disease prevention and clinical care, yet nutrition training in medical education is often limited and may not adequately prepare future physicians for dietary counselling. Valid assessment tools are therefore needed to identify specific educational gaps and guide curriculum improvement. Unlike general nutrition knowledge instruments, the present questionnaire was specifically developed for medical students and integrates factual nutrition knowledge, applied food composition knowledge, and dietary habits within a multidimensional assessment framework. This study aimed to develop and preliminarily validate a multidimensional questionnaire assessing nutrition knowledge, food composition knowledge, and dietary habits in medical students. **Methods**: A cross-sectional psychometric validation study with a mixed-methods approach was conducted at the University of Santiago de Compostela, Spain. The instrument was developed by the research team using the revised General Nutrition Knowledge Questionnaire by Kliemann et al. as a conceptual reference and was subsequently adapted to the educational context and expected baseline knowledge of medical students. Face validity was examined through student focus groups, and content validity was established by an expert panel. The initial questionnaire included 61 analyzable items across three domains. Internal consistency was evaluated using Cronbach’s alpha, construct validity by exploratory factor analysis with Promax rotation, and sensitivity analysis was performed to refine the scale. **Results**: A total of 150 medical students from all six academic years participated (mean age 21.26 years). The initial instrument showed high overall internal consistency with a Cronbach’s alpha = 0.824. After excluding 15 low-performing items, the final 46-item version demonstrated improved reliability (alpha = 0.849). Domain-specific reliability was moderate for general nutrition knowledge (alpha = 0.632) and acceptable for food composition knowledge (alpha = 0.751) and dietary habits (alpha = 0.776). The revised structure produced interpretable components with potential utility for educational diagnosis. **Conclusions**: The refined questionnaire showed preliminary psychometric validity as a multidimensional assessment tool for medical students. Its potential value lies in providing a framework for future assessment of nutrition-related educational needs rather than in directly producing curricular or behavioral change. Independent multicenter and cross-cultural validation is required before broader use can be recommended.

## 1. Introduction

Non-communicable diseases remain one of the major public health challenges worldwide and account for a substantial proportion of global morbidity and mortality [1]. Among modifiable risk factors, diet plays a particularly important role, with dietary risks estimated to contribute to approximately one in five deaths and one in six disability-adjusted life years globally [2]. Nutrition is therefore a central component of disease prevention, health promotion, and clinical care. Dietary behavior is influenced by a wide range of factors, including family and social environment, access to reliable health information, motivation, beliefs about nutrition, exposure to misinformation, and the ability to critically appraise health-related messages. In this context, knowledge, attitudes and practices studies are useful for identifying educational needs, misconceptions and behavioral patterns withing specific population, thereby informing the design of targeted interventions [3].

Healthcare professionals are expected to provide evidence-based dietary counselling as part of routine clinical practice [4,5]. Nevertheless, previous studies have consistently shown that nutrition education in medical curricula is often insufficient, fragmented, limited in duration, and poorly integrated into clinical training [6,7,8,9]. This educational gap may undermine medical students’ and physicians’ confidence in delivering nutrition advice and may ultimately affect the quality of patient care. The issue is also relevant at the individual level, as medical students are not only future providers of dietary counselling but are also subject to the same social, informational, and commercial influences that shape dietary behavior in the general population. Previous research has suggested that greater nutrition knowledge may be associated with healthier lifestyle behaviors, including improved diet quality, higher levels of physical activity, lower tobacco use, and less harmful alcohol consumption [10,11,12,13]. Medical students constitute a heterogeneous educational population, and their nutrition-related knowledge and confidence may vary according to their stage of training and previous curricular exposure [6,11,12]. The present study therefore considered students from all six academic years of the medical degree, encompassing different levels of undergraduate medical training. The questionnaire was not intended to assess knowledge acquired in a specific subject, but rather to identify common nutrition-related educational needs across different stages of the degree. Dietary habits were considered a distinct dimension, as previous studies have shown that nutrition knowledge and educational exposure may be associated with dietary and other health-related behaviors among medical and university students [10,11,12,13]. Although several instruments have been developed to assess nutrition knowledge, the General Nutrition Knowledge Questionnaire (GNKQ), originally proposed by Parmenter and Wardle and later revised by Kliemann et al., remains one of the most widely used [14,15]. However, this instrument was primarily designed for the general population and may not fully capture the specific educational profile of medical students, who are expected to have a higher baseline level of knowledge, distinct learning needs, and a future professional role in nutrition counselling [15,16,17].

In the present study, a multidimensional approach refers to the assessment of several related but conceptually distinct domains rather than to a single overall latent construct. Specifically, the questionnaire assesses general nutrition knowledge, food composition knowledge, and dietary habits separately. This approach is consistent with multidomain nutrition knowledge instruments such as the GNKQ and its revised version [14,15], as well as with broader Knowledge, Attitudes and Practices (KAP) frameworks used to examine different dimensions of health-related knowledge and behavior [3]. Domain-specific interpretation was therefore considered more informative than relying on a single global score.

## 2. Materials and Methods

### 2.1. Study Design and Setting

**Figure 1:**
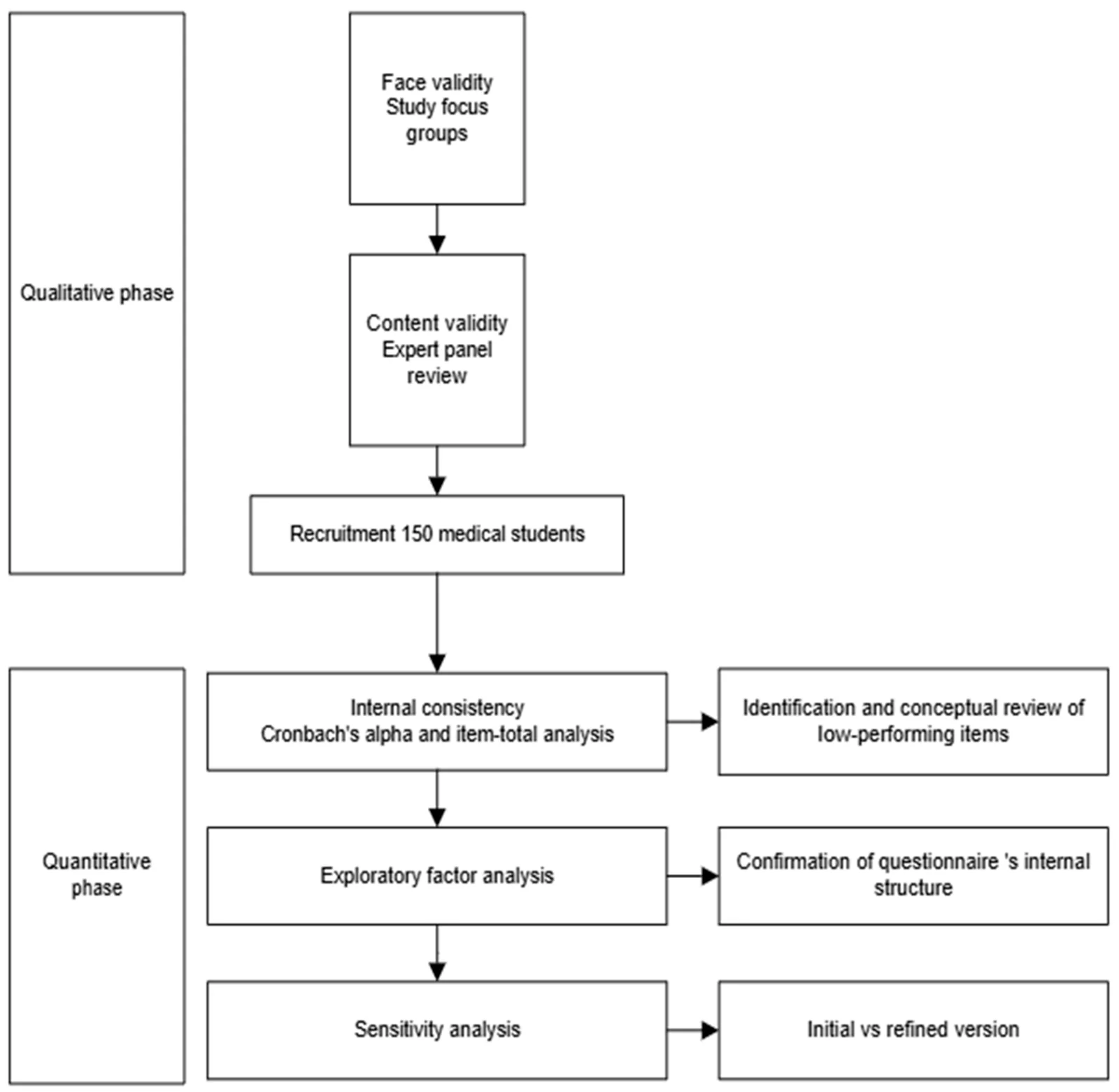
Validation process.

A cross-sectional psychometric validation study with a mixed-methods design was conducted between September 2025 and May 2026 in Santiago de Compostela, Spain. The target population comprised undergraduate students enrolled in the Degree in Medicine at the University of Santiago de Compostela during the 2025/2026 academic year. The validation process was carried out in two sequential phases. First, a qualitative phase was undertaken to assess face and content validity. Second, a quantitative phase was performed to examine internal consistency, exploratory construct validity, and the effect of item refinement on the psychometric performance of the instrument. The validation process therefore comprised three main stages, as can be seen in Figure 1: initial questionnaire development by the research team, qualitative refinement through student focus groups and expert review, and field administration followed by quantitative psychometric evaluation.

### 2.2. Questionnaire Development and Structure

The questionnaire was developed by the research team using the GNKQ as a conceptual reference, particularly the original instrument described by Parmenter and Wardle [14] and the revised version proposed by Kliemann et al. [15]. The instrument was adapted to the educational context and professional profile of medical students, taking into account that participants could be at different stages of the six-year medical degree and therefore have different levels of previously acquired knowledge. The questionnaire was designed to assess core nutrition-related domains across academic years rather than knowledge linked to a specific course or subject.

The initial questionnaire was organized into four sections (Table 1). Section 1 collected sociodemographic and contextual data. The remaining three sections comprised 61 analyzable items distributed across the domains of general nutrition knowledge, food composition knowledge, and dietary habits. The instrument was designed as a multidimensional questionnaire rather than on a single global scale.

In Section 2 and Section 3, responses were recorded on five-point Likert scales ranging from “strongly agree” to “strongly disagree”. Section 4 used a five-category frequency scale ranging from “every day” to “never”. Response options were coded from 1 to 5 according to their concordance with scientific evidence and expert consensus. Reverse-worded items were recorded so that higher scores consistently reflected greater agreement with nutritional recommendations or healthier behaviors [18,19]. The complete refined questionnaire, including final item wording and response options, is provided in Supplementary Table S1.

### 2.3. Participants and Sample Size

Students were eligible to participate if they were aged 18 years or older and were enrolled in any of the six academic years of the Degree in Medicine at the University of Santiago de Compostela during the 2025–2026 academic year.

Sample size was determined according to commonly accepted recommendations for exploratory psychometric studies, including a ratio of approximately 5–10 participants per item within each subscale and a minimum sample size of 100 participants [20,21]. On this basis, a final sample of 150 students was considered adequate for this preliminary validation phase.

### 2.4. Qualitative Validation

Face validity was assessed through three focus groups comprising 23 medical students (7, 8, and 8 participants, respectively), with students from all six academic years represented. Participants reviewed the questionnaire item by item in terms of wording, clarity, interpretability, organization, and perceived relevance. Suggestions were discussed within the groups and subsequently reviewed by the research team, and agreed modifications were incorporated into the instrument.

Content validity was subsequently evaluated by a multidisciplinary expert panel with expertise in nutrition, medical education, public health, research methodology, and psychometrics. The panel reviewed item relevance, clarity, comprehensiveness, and conceptual adequacy in relation to the constructs under study [22]. This assessment was based on qualitative expert consensus; no formal quantitative content-validity index was calculated.

### 2.5. Data Collection

Data were collected between February and April 2026 using an online questionnaire administered through Microsoft Forms [23]. Students were invited to participate through academic WhatsApp groups [24], QR codes displayed in classrooms, and posters placed at the Faculty of Medicine. Participation was voluntary, anonymous, and uncompensated.

### 2.6. Quantitative Validation and Statistical Analysis

Descriptive statistical analyses were performed using IBM SPSS Statistics for Windows, version 19.0 (IBM Corp., Armonk, NY, USA) [25]. Continuous variables were summarized as means, standard deviations (SDs), and 95% confidence intervals (CIs), whereas categorical variables were described using absolute and relative frequencies. Missing data were reviewed before analysis. No imputation was performed. Participants with missing responses in a given section were excluded only from the analyses of that section.

Internal consistency was assessed using Cronbach’s alpha [26,27]. Given the multidimensional nature of the questionnaire, reliability coefficients were calculated separately for Section 2, Section 3, and Section 4, as well as globally across the three non-demographic sections. In the interpretation of results, section-specific coefficients were prioritized over the overall coefficient.

Item-level performance was examined using corrected item-total correlations, squared multiple correlations, and Cronbach’s alpha if the item was deleted. Items with corrected item-total correlations <0.20 were considered candidates for psychometric and conceptual review.

Exploratory factor analysis was performed separately for each refined non-demographic section. Given the ordinal five-category response format, polychoric correlation matrices were used. Factors were extracted using principal axis factoring. The number of factors retained was determined by parallel analysis based on 10,000 simulated datasets using the 95th percentile criterion, together with scree plot inspection and conceptual interpretability. McDonald’s omega total was calculated as a complementary reliability indicator. Absolute factor loadings ≥0.40 were considered salient. Cross-loading was defined as an absolute loading ≥0.30 on two or more factors with a difference of <0.20 between the two highest loadings. Item communalities were also examined to assess the proportion of variance in each item accounted for by the retained factor solution. These psychometric analyses were performed in Python 3.13 using custom scripts based on pandas, NumPy, and SciPy. For multifactor solutions, Promax oblique rotation was applied, and the reported loadings correspond to the pattern matrix. Pattern coefficients were used for factor interpretation and for identifying salient and cross-loadings [28].

A sensitivity analysis was conducted to assess the effect of removing low-performing items. Items were excluded only when three conditions were simultaneously met: a corrected item-total correlation below 0.20, unfavorable psychometric performance, and absence of meaningful loss of content validity.

### 2.7. Ethical Considerations

The study was conducted in accordance with the Declaration of Helsinki and Spanish data protection legislation [29]. Ethical approval was granted by the Santiago-Lugo Research Ethics Committee on 23 July 2025 (registration code 2025/320). Data collection began only after approval had been obtained. Participation was voluntary and anonymous, and completion of the questionnaire was considered to imply informed consent.

## 3. Results

### 3.1. Face and Content Validity

The three focus groups identified several opportunities to improve item clarity, questionnaire organization, and content coverage. Wording and formatting were revised to improve comprehension and visual organization. Participants also suggested additional sociodemographic and nutrition-related content; as a result, five contextual or sociodemographic items and four nutrition-related items were incorporated. Within the dietary habits section, one item considered insufficiently informative was removed and another was divided into two separate items to distinguish different behaviors.

The resulting version was subsequently reviewed by the expert panel, which considered the included items and their organization to provide adequate comprehensibility, relevance, and conceptual coverage for field administration.

### 3.2. Sample Characteristics

A total of 150 medical students participated in the study. The mean age was 21.26 years (SD = 2.33), and 72.7% of participants identified as women. Most students had a calculated BMI within the normal-weight range (80.0%).

Social media and general media were the most frequently reported sources of nutrition information (62.7%), followed by the medical curriculum (48.0%) and the social environment (40.7%). Only 39.3% of participants reported being currently able to provide nutritional advice to patients, whereas 50.0% expected to develop this competence after graduation.

Continuous and categorical characteristics of the sample are presented in Table 2 and Table 3, respectively.

Among students reporting supplement use in the previous six months (43.3%), the most common motivations were general health and disease prevention (49.2%) and sports performance (47.7%). Less frequent reasons included medical prescription for deficiency correction (18.5%) and academic performance (12.3%) (Table 3).

### 3.3. Internal Consistency of the Initial Version

No missing data were found in the general nutrition knowledge or food classification sections. In the dietary habits section, three participants (2.0%) had incomplete responses. Therefore, Section 2 and Section 3 were analyzed using all 150 participants, whereas Section 4 and the combined analyses included 147 participants.

The initial 61-item questionnaire demonstrated high overall internal consistency across the three non-demographic sections (Cronbach’s alpha = 0.824). However, reliability varies across domains.

The general nutrition knowledge section showed moderate internal consistency (Cronbach’s alpha = 0.576), whereas the food classification (Cronbach’s alpha = 0.702) and dietary habits (Cronbach’s alpha = 0.748) sections showed acceptable reliability.

Item performance was examined separately within each domain. Fifteen items showed corrected item–total correlations below 0.20 and were therefore subjected to additional statistical and conceptual review. These items were removed only when low item–total association was accompanied by unfavorable psychometric performance and their exclusion was considered not to compromise content coverage. The item-level results supporting these decisions are presented in Table 4.

### 3.4. Sensitivity Analysis and Refined Version

Fifteen items showed corrected item-total correlations below 0.20 and were reviewed conceptually and statistically. Their removal did not compromise content validity and improved reliability indicators.

The refined questionnaire contained 46 non-demographic items: 11 assessing general nutrition knowledge, 18 assessing food composition knowledge, and 17 assessing dietary habits. Item refinement improved internal consistency and mean inter-item correlations, although improvements in factorial structure were not demonstrated for all sections.

McDonald’s omega total was calculated as a complementary indicator of internal consistency. The refined general nutrition knowledge section showed a McDonald’s omega of 0.717, compared with a Cronbach’s alpha of 0.632. The food classification and dietary habits sections showed higher reliability, with omega coefficients of 0.870 and 0.875, respectively, compared with Cronbach’s alpha values of 0.751 and 0.776. Although omega coefficients were consistently higher than the corresponding alpha coefficients, these results were interpreted together with the dimensionality and factor-loading patterns of each section rather than as isolated evidence of reliability.

A comparison between initial and refined versions is shown in Table 5.

Following refinement, Cronbach’s alpha increased from 0.824 to 0.849 for the overall non-demographic item set and from 0.576 to 0.632, 0.702 to 0.751, and 0.748 to 0.776 for general nutrition knowledge, food composition knowledge, and dietary habits, respectively. Mean inter-item correlations also increased in all three domains, indicating greater internal homogeneity after item removal.

### 3.5. Exploratory Construct Validity

Exploratory factor analysis was conducted separately for the three refined non-demographic sections using polychoric correlation matrices. The Kaiser–Meyer–Olkin (KMO) values were 0.667 for general nutrition knowledge, 0.688 for food composition knowledge, and 0.686 for dietary habits. Bartlett’s tests of sphericity were statistically significant for all three sections: general nutrition knowledge, χ^2^(55) = 283.13, *p* < 0.001; food composition knowledge, χ^2^(153) = 945.89, *p* < 0.001; and dietary habits, χ^2^(136) = 951.26, *p* < 0.001. These results supported the suitability of the correlation matrices for exploratory factor analysis.

Complete factor-loading matrices for the three refined sections are presented in Supplementary Tables S2–S4. In the general nutrition knowledge section, factor loadings ranged from 0.296 to 0.574, and seven of the eleven items reached the predefined salient-loading threshold of 0.40. Four items showed lower loadings: polyunsaturated fats as more harmful than saturated fats (0.296), whole milk as preferable because of its calcium content (0.307), use of the hand method for a balanced diet (0.314), and Mediterranean/Atlantic diets as balanced dietary patterns (0.334). Communalities ranged from 0.088 to 0.330, with a mean of 0.197. The single retained factor explained 19.7% of the total variance.

For food composition knowledge, the three-factor solution explained 37.4% of the total variance, and 14 of the 18 items showed absolute factor loadings ≥0.40. The factors were interpreted as recognition of healthy and protein- or fiber-rich foods, identification of processed foods high in salt or added sugar, and rejection of false nutritional attributions.

For dietary habits, the three-factor solution explained 42.0% of the total variance, with 14 of the 17 items reaching the predefined loading threshold. The factors were interpreted as avoidance of unhealthy behaviors, healthy dietary patterns and food self-management, and meal regularity. No items in the multidimensional sections met the predefined cross-loading criterion.

Parallel analysis supported the retention of one factor for the general nutrition knowledge section and three factors for both the food classification and dietary habits sections. The single factor retained for general nutrition knowledge explained 19.7% of the total variance. Factor loadings ranged from 0.296 to 0.574, with seven of the eleven items reaching the predefined salient-loading threshold of 0.40. Item communalities ranged from 0.088 to 0.330, with a mean communality of 0.197.

The three-factor solution for food composition knowledge explained 37.4% of the total variance. The factors were conceptually interpreted as recognition of healthy and protein- or fiber-rich foods, identification of processed foods high in salt or added sugar, and rejection of false nutritional attributions. Fourteen of the eighteen items showed salient loadings of at least 0.40.

The three-factor solution for dietary habits explained 42.0% of the total variance. The retained factors were interpreted as avoidance of unhealthy behaviors, healthy dietary patterns and food self-management, and meal regularity. Fourteen of the seventeen items reached the predefined loading threshold. No items in the food classification or dietary habits sections met the predefined criterion for relevant cross-loading. Complete factor-loading matrices are provided in the Supplementary Materials (Table S1).

All sections showed acceptable sampling adequacy and statistically significant Bartlett’s tests, supporting the appropriateness of exploratory factor analysis (Table 6).

## 4. Discussion

To the best of our knowledge, this is the first study to develop and preliminarily validate a multidimensional questionnaire on food and nutrition specifically designed for undergraduate medical students at the University of Santiago de Compostela. The study addresses a clinical and public health-relevant issue, as unhealthy dietary patterns are among the main modifiable determinants of non-communicable disease burden [1,2]. In this context, KAP-derived instruments can help identify knowledge gaps, beliefs, and behavioral patterns and thereby support more targeted educational interventions [3].

The validation process combines qualitative and quantitative methods. Student focus groups and expert review were used to assess item comprehension, relevance, and content adequacy, followed by psychometric evaluation. This approach is appropriate during early instrument development because validity depends not only on statistical performance but also on whether items are understandable, relevant, and consistently interpreted by the target population [22,23].

The findings support the multidimensional nature of the questionnaire. Rather than functioning as a single scale, the instrument assesses distinct but related domains, including general nutrition knowledge, food composition knowledge, dietary habits, perceived competence, and personal contextual factors. Accordingly, section-specific interpretation is more appropriate than relying on a single overall score, particularly for multidimensional instruments [22,26,27].

The initial 61-item version showed high overall internal consistency, with a Cronbach’s alpha of 0.824. After removing 15 items with low corrected item–total correlations, the refined 46-item version improved overall reliability to 0.849. Section-specific alpha values were 0.632 for general nutrition knowledge, 0.751 for food classification, and 0.776 for dietary habits. These findings suggest that item refinement improved the homogeneity of the instrument, although the resulting structure should still be considered preliminary rather than definitive.

The general nutrition knowledge section showed the weakest psychometric performance. Although McDonald’s omega was 0.717, compared with a Cronbach’s alpha of 0.632, the higher omega coefficient should not be interpreted as evidence of a robust unidimensional scale. The exploratory factor analysis retained a single factor, but this factor explained only 19.7% of the total variance. In addition, the mean communality was 0.197, and four of the eleven items did not reach the predefined loading threshold of 0.40. Taken together, these findings indicate limited common variance and weak internal homogeneity within this section.

This result may partly reflect the conceptual breadth of the general nutrition knowledge domain. Its items address relatively diverse topics, including dietary recommendations, nutrients, body mass index, gluten, fiber, light products, food labelling, and nutritional misconceptions. Knowledge of one of these topics does not necessarily imply knowledge of the others; therefore, strong correlations between all items should not automatically be expected. Nevertheless, conceptual heterogeneity alone does not eliminate the reliability and construct-validity concerns identified in this study. Cronbach’s alpha and McDonald’s omega must be interpreted alongside the factor loadings, communalities, and percentage of explained variance rather than as isolated coefficients [26,27,30].

The discrepancy between alpha and omega may also reflect the restrictive assumptions underlying Cronbach’s alpha, particularly tau-equivalence, which may not be met when items contribute unequally to a construct. However, the omega value of 0.717 does not compensate for the low explained variance and weak communalities observed in this section. Consequently, the general nutrition knowledge score should be interpreted cautiously and should currently be used primarily for exploratory, group-level educational assessment rather than individual-level classification. Further development of this domain may require a clearer conceptual organization. Future versions could divide general nutrition knowledge into narrower subdomains, such as evidence-based dietary recommendations, interpretation of food products and labels, basic technical nutrition concepts, and identification of nutritional misconceptions. Alternatively, additional items could be developed to provide more complete coverage of each proposed domain. These possibilities should be examined in an independent sample before changes are made to the current version, as further data-driven item refinement in the same sample would increase the risk of overfitting.

The food composition knowledge section showed more favorable psychometric performance. Although it was originally organized around four nutrient-based domains (salt, added sugar, protein, and fiber) the empirical structure did not exactly reproduce this theoretical organization. The three-factor solution was nevertheless conceptually interpretable. The first factor primarily comprised items assessing recognition of protein- and fiber-rich foods, whereas the second grouped items concerning processed foods high in salt or added sugar. The third factor mainly included items requiring rejection of incorrect nutrient-content attributions. These patterns suggest that participants’ knowledge of food composition may be organized around broader food and nutrient associations rather than strictly separated nutrient-specific categories. However, these interpretations remain exploratory and require confirmation in an independent sample [3].

The dietary habits section showed the most stable psychometric performance. Its internal structure was conceptually interpretable and reflected three broad dimensions: avoidance of unhealthy habits, healthy dietary patterns and food self-management, and meal regularity. This is consistent with the way lifestyle behaviors tend to occur in practice, as clusters rather than isolated behaviors. Previous studies have also suggested that greater nutrition knowledge may be associated with healthier dietary and lifestyle patterns among university students and health-related populations [10,11,12,13,17]. In contrast, the food classification and dietary habits sections showed more favorable reliability, with McDonald’s omega coefficients of 0.870 and 0.875, respectively. These values, together with their interpretable three-factor structures and higher percentages of explained variance, provide stronger preliminary support for these sections. However, because omega total can also be influenced by multidimensionality, these coefficients should be interpreted in conjunction with the factor structure rather than as evidence that each section constitutes a single homogeneous scale.

Removing 15 poorly performing items increased overall, as well as section-specific internal consistency and mean inter-item correlations. However, these improvements did not necessarily translate into stronger factorial performance, particularly for the general nutrition knowledge section. However, item removal should not be based exclusively on statistical criteria, because some items with low correlations may still be educationally or conceptually relevant. In this study, refinement considered both psychometric behavior and preservation of content validity, in line with recommendations for instrument development and validation [22,23].

The refined version is shorter, more homogeneous, and potentially easier to use in future educational or research settings. Nevertheless, because item refinement and reanalysis were conducted in the same sample, overfitting cannot be excluded. External validation in an independent population is therefore required before the instrument can be considered definitive.

The questionnaire was developed with reference to the GNKQ-R and its predecessor [14,15], both widely used and adapted across different populations and settings [15,16,17]. However, the present instrument was not a simple translation. Its content, organization, and response format were adapted to the educational context and expected baseline knowledge of medical students. Because the present questionnaire is newly developed, no previous studies have used the identical instrument; comparisons with the existing literature therefore concern related nutrition knowledge questionnaires and the broader assessment of nutrition competencies rather than direct comparison of questionnaire scores.

The results are also consistent with previous evidence showing that nutrition education in undergraduate medical programs is frequently insufficient, fragmented, and poorly integrated into clinical practice, despite the important role of physicians in dietary counselling and chronic disease prevention [4,5,6,7,8,9]. This reinforces the need for specific tools capable of identifying educational gaps among medical students.

One of the most relevant descriptive findings was that media and social networks were the most frequently reported sources of nutrition information, ahead of the medical curriculum. Although this result should be interpreted cautiously, it may reflect an important educational gap. In an environment characterized by widespread exposure to contradictory and non-evidence-based nutritional information, medical schools should ideally be a major source of rigorous scientific knowledge.

Likewise, only a proportion of students perceived themselves as currently capable of providing nutritional counselling. This is important because theoretical knowledge alone does not ensure that future physicians feel prepared to address nutrition with patients. Effective dietary counselling requires up-to-date knowledge, critical appraisal skills, communication abilities, and the capacity to adapt recommendations to individual clinical and social circumstances [4,5].

The potential educational value of this questionnaire lies in its use as a first step in a needs-assessment process rather than as an educational intervention in itself. Once externally validated, domain scores could be examined across academic years and mapped against existing nutrition-related curricular content. Areas showing consistently lower performance could then inform targeted educational strategies, such as dedicated nutrition teaching, case-based learning, food-label interpretation, or dietary counselling training, in line with recommendations advocating greater integration of nutrition into medical education and a competency-based approach to dietary counselling [4,5]. Subsequent studies should evaluate whether such interventions actually improve students’ knowledge and competencies. Similarly, findings related to dietary habits may help generate hypotheses for student health-promotion initiatives, although changes to the university food environment would require separate assessment.

From a public health perspective, this is particularly relevant because unhealthy diet is a major contributor to the burden of chronic disease [1,2]. Physicians in Primary Care, Preventive Medicine, Endocrinology, Cardiology, Gastroenterology, and other specialties play an important role in promoting healthy behaviors. Medical education should therefore ensure that future physicians acquire sufficient nutrition knowledge and communication skills. Although the present instrument is specifically designed for medical students and does not assess patient adherence or post-treatment health education, strengthening nutrition competencies during medical training may ultimately improve the quality of dietary counselling provided to patients.

### Strengths and Limitations

This study has several strengths. First, it used a mixed-methods approach combining student focus groups, expert review, and quantitative psychometric analysis, thereby assessing both conceptual adequacy and statistical performance [22,23]. Second, the questionnaire was analyzed according to its multidimensional structure rather than as a single global scale, which is particularly appropriate for a KAP-derived instrument [3]. Third, the sensitivity analysis allowed the proposal of a shorter and more homogeneous version, with refinement based not only on Cronbach’s alpha but also on corrected item–total correlations, overall psychometric performance, and preservation of content validity [22,23,26,30]. Finally, exploratory factor analysis was conducted separately for each section, and component retention was based on complementary criteria, including parallel analysis, which is more robust than relying exclusively on eigenvalues greater than one [21,28].

Several limitations should also be acknowledged. This was a single-center study conducted in one medical school, which limits generalizability. Although the sample of 150 students was acceptable for a preliminary exploratory validation, larger and independent samples are needed to confirm the stability of the internal structure [21]. In addition, several variables were self-reported and may therefore be affected by recall or social desirability bias. Test–retest reliability was not assessed, so the temporal stability of the questionnaire remains unknown [3].

From a psychometric perspective, the general nutrition knowledge section remains the main limitation of the instrument. Although McDonald’s omega was acceptable (ω = 0.717), the retained factor explained only 19.7% of the total variance, the mean communality was low (0.197), and four of the eleven items failed to reach the predefined loading threshold of 0.40. These findings provide limited evidence for a strong common latent dimension and indicate that this domain requires further conceptual refinement. In addition, item selection and evaluation of the refined questionnaire were performed using the same sample, which may have resulted in overfitting and optimistic estimates of psychometric performance. Therefore, the present factor structure should be considered preliminary and requires confirmation in an independent, preferably multicenter sample. The exploratory factor analysis conducted in this study should therefore be interpreted as an initial exploratory approach to the questionnaire’s internal structure [21,28].

Another important limitation is that item selection and evaluation of the refined questionnaire were conducted using the same sample. This may have introduced overfitting and resulted in optimistic estimates of reliability and factorial performance. Given the available sample size, splitting the dataset into independent development and validation subsamples was not considered appropriate. Therefore, the refined 46-item version should be regarded as a preliminary candidate instrument rather than a definitively validated questionnaire. No further items were removed on the basis of the additional exploratory factor analysis, as further data-driven refinement within the same sample could increase this risk.

External validation in a larger, independent, and preferably multicenter sample is therefore essential. Future studies should determine whether the retained items, reliability estimates, and factor structure remain stable across different medical schools and educational settings, and should include confirmatory factor analysis and test–retest reliability before the questionnaire is recommended for broader use. In addition, questionnaire domains should be compared across academic years and related to students’ previous nutrition-related coursework. Such analyses would help identify whether specific knowledge or competency gaps are concentrated at particular stages of medical training and could provide a more direct basis for targeted curricular modifications. This approach is especially relevant because nutrition education among future healthcare professionals is widely recognized as an area requiring improvement, while the specific curricular changes needed remain insufficiently defined [4,5,6,7,8,9].

Overall, the findings support the preliminary usefulness of the questionnaire as a multidimensional tool for assessing nutrition-related knowledge, food composition knowledge, and dietary habits among medical students. Its main contribution lies in its potential use as a diagnostic instrument to identify educational gaps and inform targeted curriculum improvements in an area of major clinical and public health relevance.

## 5. Conclusions

This study developed and preliminarily validated a multidimensional questionnaire assessing general nutrition knowledge, food composition knowledge, and dietary habits among medical students. The refined instrument provides a preliminary measurement framework rather than an educational intervention in itself. If its psychometric properties are confirmed in independent multicenter samples, it could be used to examine nutrition-related needs across academic years, relate identified weaknesses to curricular exposure, and support the design of targeted educational strategies whose effectiveness should subsequently be evaluated. The current 46-item version should therefore be considered a preliminary candidate instrument requiring further external validation before broader implementation.

## Figures and Tables

**Table 1:** General structure of questionnaire.

Section	Content	Variable Type	Analytical Purpose
Section 1. Sociodemographic and personal context	Gender, age, height, weight, chronic disease, degree, university, academic year, residence, diet, supplement use, sources of nutrition knowledge, perceived ability to provide dietary counselling, household income	Categorical, ordinal, continuous, and free-text variables	Sample description and explanatory variables
Section 2. General nutrition knowledge	15 items on dietary recommendations, nutrients, body mass index (BMI), gluten, fiber, light products, sports drinks, food labelling, Mediterranean/Atlantic diet	5-point Likert scale	Main general nutrition knowledge section
Section 3. Food classification	26 items on salt, added sugar, protein, and fiber content	5-point Likert scale	Applied knowledge/food classification section
Section 4. Dietary habits	20 items on breakfast, meals, water, soft drinks, alcohol, meat, fish, fruit, vegetables, dairy products, fast food, cooking, food labels, processed foods, physical activity, and tobacco	Ordinal frequency scale	Dietary habits and healthy lifestyle profile

**Table 2:** Continuous sample characteristics.

Variable	n	Mean ± SD	95% CI
Age, years	150	21.26 ± 2.33	20.89–21.63
Height, m *	150	1.69 ± 0.10	1.68–1.71
Weight, kg **	150	63.83 ± 11.36	62.01–65.65
Calculated BMI ***, kg/m^2^	150	22.17 ± 2.63	21.75–22.59

* Meters, ** Kilograms, *** Body Mass Index.

**Table 3:** Main categorical sample characteristics.

Variable	n (%)
Gender	
Women	109 (72.7)
Men	39 (26.0)
Non-binary	2 (1.3)
Academic year with most credits enrolled	
1st year	37 (24.7)
2nd year	28 (18.7)
3rd year	26 (17.3)
4th year	18 (12.0)
5th year	20 (13.3)
6th year	21 (14.0)
Calculated BMI * category	
Underweight	7 (4.7)
Normal weight	120 (80.0)
Overweight	21 (14.0)
Obesity	2 (1.3)
Perceived-calculated BMI * concordance	
Concordant	130 (86.7)
Weight under perception	15 (10.0)
Weight over perception	5 (3.3)
Supplement use in previous 6 months	
No	85 (56.7)
Yes	65 (43.3)
Main sources of nutrition information	
Media and social networks	94 (62.7)
Medical curriculum	72 (48.0)
Family/friends/personal environment	61 (40.7)
Scientific literature self-learning	52 (34.7)
Professional dietary counselling	18 (12.0)
Non-formal training	16 (10.7)
No nutrition training	14 (9.3)
Other degrees/cycles	6 (4.0)
Other	2 (1.3)
Perceived ability to provide nutritional advice	
Yes, currently able	59 (39.3)
Not yet, but expect to be able after graduation	75 (50.0)
Do not expect to be able after graduation	16 (10.7)
Reasons for supplement use	
General health and prevention	32 (49.2)
Sports performance	31 (47.7)
Professional prescription to correct a deficiency	12 (18.5)
Academic performance	8 (12.3)
Compensation for dietary habits	4 (6.2)
Other	2 (3.1)
Aesthetics or weight control	1 (1.5)

* Body Mass Index.

**Table 4:** Items removed in the refined version.

Section	Removed Item	Corrected Item-Total Correlation	Alpha If Item Deleted
Section 2	Oily fish 1–2 times/week	0.018	0.589
Section 2	Drinking 2 L of water/day	−0.062	0.596
Section 2	Reading food labels when shopping	0.107	0.577
Section 2	Sports drinks after exercise	0.086	0.587
Section 3	Breakfast cereals as high in salt	0.079	0.708
Section 3	Bread as high in salt	−0.063	0.718
Section 3	Fabada as high in salt	0.110	0.702
Section 3	Light cola drink as high in added sugar	0.128	0.706
Section 3	Butter as protein-rich	0.175	0.699
Section 3	Nuts as protein-rich	0.138	0.703
Section 3	Banana as fiber-rich	0.084	0.707
Section 3	White rice as fiber-rich	0.194	0.700
Section 4	Eating five meals per day	0.131	0.756
Section 4	Drinking 2 L of water/day	0.082	0.758
Section 4	Eating fish	0.017	0.754

**Table 5:** Comparison between initial and refined versions.

Variable	Global	Section 2	Section 3	Section 4
Valid n (initial)	147	150	150	147
Valid n (refined)	147	150	150	147
No. of items (initial)	61	15	26	20
No. of items (refined)	46	11	18	17
Cronbach’s alpha (initial)	0.824	0.576	0.702	0.748
Cronbach’s alpha (refined)	0.849	0.632	0.751	0.776
McDonald’s omega total (refined)	-	0.717	0.870	0.875
Standardized alpha (initial)	0.836	0.567	0.736	0.754
Standardized alpha (refined)	0.854	0.639	0.766	0.784
Mean inter-item correlation (initial)	0.077	0.080	0.097	0.133
Mean inter-item correlation (refined)	0.113	0.139	0.154	0.176
Alpha difference after refinement	+0.025	+0.055	+0.049	+0.028

**Table 6:** Construct validity indicators: initial and refined versions.

Variable	Section 2	Section 3	Section 4
No. of items (initial)	15	26	20
No. of items (refined)	11	18	17
KMO (initial)	0.619	0.636	0.733
KMO (refined)	0.667	0.688	0.686
Bartlett chi-square (initial)	236.49	818.03	672.66
Degrees of freedom (initial)	105	325	190
*p*-value (initial)	<0.001	<0.001	<0.001
Bartlett chi-square (refined)	283.13	945.89	951.26
Degrees of freedom (refined)	55	153	136
*p*-value (refined)	<0.001	<0.001	<0.001
Components retained (initial)	3	4	3
Explained variance (initial)	36.5%	37.7%	40.3%
Components retained (refined)	1	3	3
Explained variance (refined)	19.7%	37.4%	42.0%

KMO, Kaiser–Meyer–Olkin measure; factor retention was based primarily on parallel analysis. Absolute factor loadings ≥0.40 were considered salient.

## Data Availability

The data presented in this study are available from the corresponding author upon reasonable request. The data are not publicly available because they contain individual-level information from a small, single-centre student population, and unrestricted public disclosure could compromise participant confidentiality. Access may be granted for legitimate scientific purposes, subject to appropriate ethical and data-protection safeguards.

## Supplementary Materials

The following supporting information can be downloaded at https://www.mdpi.com/article/10.3390/nu18162720/s1, Table S1. Refined questionnaire (Spanish version). Table S2. Factor-loading matrix for the refined general nutrition knowledge section. Table S3a. Rotated factor-loading matrix for the refined food Composition Knowledge section. Table S3b. Factor correlated matrices for the refined food Composition Knowledge section. Table S4a. Rotated factor-loading matrix for the refined dietary habits section. Table S4b. Factor correlated matrices for the refined dietary habits section.
