# Supplementary material for: Development and Preliminary Validation of a Multidimensional Questionnaire on Nutrition Knowledge, Food Composition Knowledge, and Dietary Habits in Medical Students
Source: Nutrients. 2026 Aug 20;18(16):2720. doi: 10.3390/nu18162720 (PMC13516349; doi:10.3390/nu18162720)
Supplement: Supplementary file 1 [file nutrients-18-02720-s001.zip › nutrients-4471201-supplementary.pdf]

Supplementary Material Table S1. Refined questionnaire (Spanish version)

Cuestionario de CONOCIMIENTOS GENERALES EN NUTRICIÓN Y ALIMENTACIÓN en el estudiantado de Medicina

Este es un cuestionario para la realización de una investigación sobre los conocimientos sobre alimentación del estudiantado de Medicina.

Al rellenar este cuestionario, usted está aceptando participar en el estudio. La cumplimentación de este cuestionario le tomará aproximadamente 10 minutos de su tiempo.
La participación en este estudio es estrictamente voluntaria. En ningún momento será requerida ningún tipo de información identificativa por su parte, siendo la cumplimentación del cuestionario totalmente anónima y recopilándose únicamente las respuestas a las preguntas siguientes.
Muchas gracias por su tiempo y colaboración.

Sección 1: Datos sociodemográficos y contextuales

1. Género:
   • Hombre
   • Mujer
   • No binario
   • Prefiero no contestar
   • Otro: ______________________________

2. Edad: ________ años

3. Talla: ________ m

4. Peso: ________ kg

5. Indique si padece o ha padecido alguna enfermedad crónica relevante (celiaquía, diabetes, depresión…):
   • ____

6. Indique el grado en el que se ha matriculado este curso: ______________________________

7. Indique la universidad en la que se ha matriculado: _________________________________

8. ¿En qué curso se ha matriculado de mayor cantidad de créditos? ______________________

9. Actualmente, ¿dónde reside?
   • Residencia familiar rural
   • Residencia familiar urbana
   • Residencia de estudiantes
   • Piso compartido
   • Otro (especificar): ______________________________

10. ¿Cuánto tiempo lleva residiendo de esta manera?
    • Menos de 1 año
    • 1 año
    • 2 años
    • Más de 2 años

11. ¿En qué grupo cree que se encuentra?
    • Bajo peso
    • Normopeso
    • Sobrepeso
    • Obesidad

12. ¿Sigue algún tipo de dieta o patrón alimentario específico?
    • No
    • Vegetariano
    • Vegano
    • Halal
    • Kosher

• Otro (especificar): _______________________________

13. ¿Ha consumido algún suplemento en los últimos 6 meses?
• Sí
• No

14. En caso de haberlo consumido, ¿cuáles fueron los motivos principales?
• Prescripción profesional para suplir un déficit
• Salud general y prevención (refuerzo del sistema inmune, bienestar general…)
• Compensación de hábitos dietéticos (dieta vegana, comidas desequilibradas…)
• Mejora del rendimiento deportivo
• Mejora del rendimiento académico
• Estética o control de peso (sustitutivos de comidas, quemagrasas…)
• Otro (especificar): _______________________________

15. ¿Cuáles son sus fuentes principales de conocimientos sobre alimentación y nutrición? Seleccione como máximo 3 opciones.
• Currículum del grado en Medicina
• Currículum de otros grados o ciclos
• Formación no reglada (cursos en plataformas, webinars no universitarios…)
• Autoaprendizaje mediante literatura científica (lectura de guías, artículos…)
• Divulgación en medios y redes sociales
• Información de personas de mi entorno
• Asesoramiento personal por un profesional (dietista, nutricionista…)
• No tengo ninguna formación
• Otro (especificar): _______________________________

16. ¿Se considera capaz de dar consejo sanitario sobre alimentación saludable a pacientes?
• Sí, me veo capaz
• Todavía no, pero creo que sí lo seré cuando termine el grado
• No creo que tenga la capacidad ni aun cuando termine el grado

17. ¿Cuál es el nivel aproximado de ingresos mensuales netos en su hogar (total de la unidad familiar)?
• Nivel bajo: menos de 1.350 €
• Nivel medio-bajo: entre 1.350 € y 2.100 €
• Nivel medio-alto: entre 2.100 € y 3.600 €
• Nivel alto: más de 3.600 €
• Prefiero no contestar / No lo sé

Sección 2: Conocimientos generales en nutrición

De acuerdo con las recomendaciones generales, indique en qué medida está de acuerdo con las siguientes afirmaciones.

	Totalmente de acuerdo	Parcialmente de acuerdo	Indiferente	Parcialmente en desacuerdo	Totalmente en desacuerdo
Un vaso de zumo sustituye a una de las piezas de fruta diarias recomendadas.	○	○	○	○	○
Las grasas poliinsaturadas son más perjudiciales que las grasas saturadas.	○	○	○	○	○
El IMC es una fuente confiable para analizar el estado nutricional.	○	○	○	○	○
Excepto el maíz y el arroz, todos los cereales contienen trazas de gluten.	○	○	○	○	○
La leche entera es mejor nutricionalmente que la desnatada al contener más calcio.	○	○	○	○	○
El riesgo de caries es el mismo al tomar una naranja o un zumo, ya que los dos contienen la misma cantidad de azúcar.	○	○	○	○	○
La ingesta de fibra es imprescindible para un buen tránsito intestinal.	○	○	○	○	○
Al aportar más energía, los lípidos son el nutriente más importante.	○	○	○	○	○
Al ser más bajos en calorías, los alimentos light son siempre una buena opción en comparación con los estándar.	○	○	○	○	○
Utilizar técnicas aproximativas (técnica de la mano, por ejemplo) para medir las cantidades de alimentos es útil para llevar una dieta equilibrada.	○	○	○	○	○
La dieta Mediterránea o la Atlántica son ejemplos de dietas equilibradas.	○	○	○	○	○

Sección 3: Conocimientos sobre composición de alimentos

	Totalmente de acuerdo	Parcialmente de acuerdo	Indiferente	Parcialmente en desacuerdo	Totalmente en desacuerdo
**Estos alimentos tienen ALTO CONTENIDO EN SAL**					
Verduras congeladas	○	○	○	○	○
Carne roja	○	○	○	○	○
Sopa precocinada	○	○	○	○	○
**Estos alimentos contienen ALTO PORCENTAJE DE AZÚCARES AÑADIDOS**					
Yogur natural	○	○	○	○	○
Helado	○	○	○	○	○
Ketchup	○	○	○	○	○
Melón	○	○	○	○	○
**Estos alimentos son ricos en PROTEÍNA**					
Ave de corral	○	○	○	○	○
Queso fresco	○	○	○	○	○
Fruta	○	○	○	○	○
Alubias	○	○	○	○	○
Tofu	○	○	○	○	○
**Estos alimentos tienen ALTO CONTENIDO EN FIBRA**					
Avena	○	○	○	○	○
Huevo	○	○	○	○	○
Pasta integral	○	○	○	○	○
Patata con piel	○	○	○	○	○
Semillas de chía	○	○	○	○	○
Brócoli	○	○	○	○	○

Sección 4: Hábitos alimentarios y relacionados con la salud

Indique la frecuencia con la que realiza las siguientes acciones:

	Todos los días	Casi todos los días	Algunos días	Casi nunca	Nunca
¿Con qué frecuencia desayuna?	○	○	○	○	○
¿Con qué frecuencia se salta una de las 3 comidas principales del día?	○	○	○	○	○
¿Con qué frecuencia bebe refrescos?	○	○	○	○	○
¿Con qué frecuencia bebe alcohol?	○	○	○	○	○
¿Con qué frecuencia consume carne roja (ternera, cerdo, cordero…)?	○	○	○	○	○
¿Con qué frecuencia consume carne procesada (embutidos, salchichas…)?	○	○	○	○	○
¿Con qué frecuencia toma cereales (pan, arroz, pasta, avena…)?	○	○	○	○	○
¿Con qué frecuencia toma fruta?	○	○	○	○	○
¿Con qué frecuencia toma verdura?	○	○	○	○	○
¿Con qué frecuencia toma productos lácteos (leche, queso, yogur…)?	○	○	○	○	○
¿Con qué frecuencia consume comida rápida?	○	○	○	○	○
¿Con qué frecuencia cocina su propia comida?	○	○	○	○	○
¿Con qué frecuencia cocina horneado o a la plancha en vez de frito?	○	○	○	○	○
¿Con qué frecuencia mira las etiquetas de los alimentos para seleccionarlos?	○	○	○	○	○
¿Con qué frecuencia toma alimentos precocinados en vez de frescos?	○	○	○	○	○
¿Con qué frecuencia realiza actividad física?	○	○	○	○	○
¿Con qué frecuencia fuma tabaco?	○	○	○	○	○

Muchas gracias por su tiempo

Table S2. Factor-loading matrix for the refined general nutrition knowledge section

Item	Factor 1	Communality
Juice replaces fruit	0.558	0.312
Polyunsaturated fats are more harmful than saturated fats	0.296	0.088
BMI is a reliable measure of nutritional status	0.520	0.270
All cereals except corn and rice contain gluten	0.574	0.330
Whole milk is preferable because it contains more calcium	0.307	0.095
Orange and orange juice have the same caries risk	0.457	0.209
Fiber is essential for intestinal transit	0.425	0.181
Lipids are the most important nutrient because they provide more energy	0.406	0.165
Light foods are always a good option	0.557	0.310
The hand method is useful for achieving a balanced diet	0.314	0.099
Mediterranean and Atlantic diets are balanced dietary patterns	0.334	0.111

Note: Exploratory factor analysis was performed using a polychoric correlation matrix and principal axis factoring. Parallel analysis supported the retention of one factor. All factor loadings are reported. Absolute factor loadings ≥0.40 were considered salient and are shown in bold. The retained factor explained 19.7% of the total variance.

Table S3a. Rotated factor-loading matrix for the refined food Composition Knowledge section

Item	Factor 1	Factor 2	Factor 3	Communality
Salt: frozen vegetables	0.300	-0.146	0.258	0.187
Salt: red meat	0.043	-0.113	0.438	0.194
Salt: ready-made soup	0.088	0.463	0.080	0.275
Added sugar: natural yogurt	0.137	-0.101	0.530	0.326
Added sugar: ice cream	-0.006	0.996	-0.014	0.982
Added sugar: ketchup	0.159	0.658	0.108	0.579
Added sugar: melon	0.174	0.079	0.431	0.297
Protein: poultry	0.480	0.206	0.264	0.515
Protein: fresh cheese	0.365	0.133	0.114	0.229
Protein: fruit	-0.170	0.236	0.698	0.540
Protein: beans	0.489	-0.132	0.061	0.237
Protein: tofu	0.561	0.032	0.195	0.443
Fiber: oats	0.370	0.177	0.221	0.331
Fiber: egg	0.051	-0.005	0.593	0.373
Fiber: wholegrain pasta	0.369	0.113	0.084	0.207
Fiber: potato with skin	0.691	0.002	-0.263	0.423
Fiber: chia seeds	0.480	0.152	0.012	0.304
Fiber: broccoli	0.502	0.095	-0.016	0.285

Exploratory factor analysis was performed using a polychoric correlation matrix, principal axis factoring, and Promax rotation. Parallel analysis supported the retention of three factors. All factor loadings are reported. Absolute factor loadings ≥0.40 were considered salient and are shown in bold. Cross-loading was defined as an absolute loading ≥0.30 on two or more factors, with a difference <0.20 between the two highest loadings; no items met this criterion. The three-factor solution explained 37.4% of the total variance.

Factor 1: Recognition of protein- and fiber-rich foods

Factor 2: Recognition of processed foods high in salt or added sugar

Factor 3: Rejection of incorrect nutrient-content attributions

Table S3b. Factor correlated matrices for the refined food Composition Knowledge section

	**F1**	**F2**	**F3**
**F1**	1.000	0.309	0.341
**F2**	0.309	1.000	0.227
**F3**	0.341	0.227	1.000

Table S4a. Rotated factor-loading matrix for the refined dietary habits section

Item	Factor 1	Factor 2	Factor 3	Communality
Breakfast	0.004	0.012	0.908	0.833
Skipping a main meal	0.089	-0.007	0.737	0.587
Soft drinks	0.559	0.168	0.066	0.416
Alcohol consumption	0.645	0.012	-0.066	0.399
Red meat consumption	0.523	-0.081	0.075	0.286
Processed meat consumption	0.531	0.167	-0.199	0.305
Cereal consumption	0.075	0.212	0.355	0.249
Fruit consumption	0.103	0.396	0.258	0.335
Vegetable consumption	0.043	0.580	0.164	0.442
Dairy product consumption	0.010	0.095	0.606	0.417
Fast-food consumption	0.747	0.117	0.082	0.661
Cooking own food	0.037	0.369	-0.009	0.142
Baking/grilling instead of frying	-0.053	0.673	0.031	0.453
Reading food labels	0.030	0.635	-0.054	0.393
Pre-cooked food consumption	0.611	-0.079	0.159	0.433
Physical activity	0.001	0.587	0.023	0.354
Tobacco use	0.645	-0.134	0.095	0.431

Note: Exploratory factor analysis was performed using a polychoric correlation matrix, principal axis factoring, and Promax rotation. Parallel analysis supported the retention of three factors. All factor loadings are reported. Absolute factor loadings ≥0.40 were considered salient and are shown in bold. Cross-loading was defined as an absolute loading ≥0.30 on two or more factors, with a difference <0.20 between the two highest loadings; no items met this criterion. The three-factor solution explained 42.0% of the total variance.
Factor 1: Avoidance of unhealthy behaviors.
Factor 2: Healthy dietary pattern and food self-management.
Factor 3: Meal regularity.

Table S4b. Factor correlated matrices for the refined dietary habits section

		F2	F3
**F1**	1.000	0.227	0.298
**F2**	0.227	1.000	0326
**F3**	0.298	0.326	1.000
